# Toxic Plants Potentially Impacting the Health Status of Livestock in St. Kitts—A Surveillance Study

**DOI:** 10.3390/toxins18090380

**Published:** 2026-09-03

**Authors:** Andras-Laszlo Nagy, Francesca Caloni, Regina Tose Kemper, Sofia Gonzalez Lleras, Heather Beigh, Christian DeJean, Maurice Matthew, Georgios Paraschou

**Affiliations:** 1 Department of Biomedical Sciences, Ross University School of Veterinary Medicine, Basseterre P.O. Box 334, Saint Kitts and Nevis; anagy@rossvet.edu.kn (A.-L.N.); regina.kemper@sruc.ac.uk (R.T.K.); sofia.gonzalez@banfield.com (S.G.L.); heather.beigh@icloud.com (H.B.); christiandejean@students.rossu.edu (C.D.); mmatthew@rossvet.edu.kn (M.M.); 2 Department of Environmental Science and Policy (ESP), Università degli Studi di Milano, Via Celoria 2, 20133 Milan, Italy; francesca.caloni@unimi.it (F.C.); 3 School of Veterinary Medicine and Biosciences, Scotland’s Rural College, Aberdeen AB21 9YA, UK; 4 Department of Comparative Biomedical Sciences, Veterinary School, University of Surrey, Guildford GU2 7XH, UK

**Keywords:** Caribbean, livestock, Phytotoxins, plants, toxicity

## Abstract

Tropical regions are characterized by exceptional biodiversity, including extremely diverse flora. Many plants produce chemical compounds that function as defense mechanisms, some of which are toxic to mammals, including livestock species. However, documentation of toxic plants in the Caribbean region remains limited. The present study aimed to identify toxic and potentially toxic plant species affecting livestock on the island of Saint Christopher (St. Kitts) through surveys of a representative number of farms across all nine parishes. An extensive literature review was performed in order to characterize the local flora and to identify plant species with known or suspected toxic potential. Farm visits were conducted using a structured surveillance questionnaire developed by the authors. During these visits, representative photographs of commonly encountered toxic plants were taken. The identified plant species were classified according to the chemical nature of their active ingredient, and their main clinical effects were described. Ten plant species (*Abrus precatorius*, *Senna occidentalis*, *Catharanthus roseus*, *Crotalaria retusa*, *Datura stramonium*, *Manihot esculenta*, *Mimosa pudica*, *Nerium oleander*, *Nicotiana tabacum* and *Ricinus communis*) were found to be widespread throughout the island and have been documented in the literature as toxic to livestock. By knowing the distribution of these species, high-risk areas and pastures can be identified, thereby supporting targeted prevention and more effective management of future plant-associated toxicosis outbreaks.

## 1. Introduction

Phytotoxins are plant-derived compounds with toxic potential and are a frequent cause of animal poisoning worldwide, causing major economic losses to the livestock industry [1,2,3]. Both livestock and pets are affected by toxic plant ingestion and the toxicological effects are influenced by the plant species and their active compounds, their growth stage, the amount ingested, and the part of the plant ingested [3]. Notably, plant toxicity is essentially dose-dependent [1].

The Caribbean is renowned for its exceptional biodiversity and it is home to a wide range of toxic plant species [4]. The variable geography and numerous habitats and microclimates sustain this extremely high diversity of plants [4]. There are not many reports regarding plant poisoning in livestock in St. Kitts and the lack of a central reporting system for animal poisonings in St. Kitts makes toxico-epidemiological study difficult. Many tropical plants contain toxic phytochemicals that function as natural defense mechanisms against herbivores, microorganisms, and parasites [5]. Apart from their toxic effect, phytochemicals have been used since antiquity by humans for their medicinal properties and have proven to be beneficial when used correctly [6,7]. However, often those chemicals are toxic to mammals [8,9,10,11,12,13,14,15,16,17]. Livestock production in St. Kitts is predominantly non-intensive and free-range, relying largely on grazing. Livestock is bred and raised to meet local demand, with sheep, goats, cattle, and swine being the most common livestock species. There is a small number of equines, with donkeys being a significant number of them, but most of the donkeys are nowadays feral. Small and large ruminants, as well as swine, are raised for their meat.

Toxic effects in livestock resulting from plant ingestion remain poorly studied in the Caribbean region.

Reports of plant poisoning in livestock are more frequently published from South America, a region that has parts that share climatic characteristics with the Eastern Caribbean. In this regard, in a comprehensive review, Riet-Correa et al. (2023) identified and evaluated 219 plant species documented as causes of livestock poisoning in South America [18]. Among the most frequently implicated taxa are members of the genus *Crotalaria*, for which numerous cases of intoxication have been reported throughout the continent [19,20]. The impetus for the present study originated from the investigation of a suspected case of *Ricinus communis* toxicosis in a goat submitted to the Necropsy Service at Ross University School of Veterinary Medicine with a history of being found dead. Gross and histopathological examination revealed lesions consistent with *R. communis* intoxication, including hepatic necrosis and ruminal epithelial degeneration [18]. However, definitive confirmation of poisoning was not possible because no plant material was identified within the gastrointestinal tract, and the owner was unable to verify exposure to pastures containing *R. communis*. Nevertheless, exposure was considered highly plausible because the herd was managed under free-range conditions and the plant is widely distributed throughout the region.

The present study surveyed livestock farms on the island of St. Kitts with the objective of identifying the presence and abundance of potentially toxic plants on pastures and around animal facilities using on-farm visits and a structured surveillance questionnaire (Supplemental Material).

The findings of this study are intended to serve as an educational tool for local veterinarians, farmers and the general public, increasing awareness of toxic plant risks and contributing to improved animal welfare by reducing the incidence of plant-related toxicoses in the Federation of St. Kitts and Nevis.

## 2. Results

The Pathology Department at Ross University School of Veterinary Medicine frequently evaluates suspected cases of plant toxicosis in livestock. In many cases, clinical histories lack relevant exposure information and associated lesions are nonspecific, complicating definitive diagnosis. As a result, plant poisoning events in St. Kitts are likely underrecognized and underreported due to the absence of structured surveillance. To address this gap, we initiated a study to document toxic and potentially toxic plant species present on the island of St. Kitts.

The study was performed in seventeen representative farms, 10 highland and 7 lowland, widespread in St. Kitts, including all parishes of the country. The farms were visited, and the presence of toxic plants was recorded. The visited farms raised multiple species. Sheep were raised on 13 farms, goats on 14, cattle on four, rabbits on one, donkeys on two, and pigs on six. The presence and distribution of toxic plants, along with chemical compounds and the location of chemical compounds, as well as whether the farm was situated in a lowland or highland, were recorded for all visited locations (Table 1).

From the initial list of 50 potentially toxic plants identified as native to the island during the literature review [6,7], 46 were recorded during our farm visits. 10 out of 46 plants were found to be toxic after ingestion by livestock (Table 2) [5,8,9,10,11,12,13,14,15,16,17,18,30,31,32,33,34,35]. From the toxic plants identified with recorded toxicity in the literature, *Ricinus communis* (Castor bean), *Mimosa pudica* (Shameful, Shame lady, Sleep lady, Sensitive plant, Touch-Me-Not), *Catharanthus roseus* (Periwinkle), and *Senna occidentalis* (Granny coffee/stinking weed/Coffee senna) were most frequently found, specifically in 15/17, 13/17, 11/17, and 10/17 farms, respectively. *Abrus precatorius* (Jumbee bead, Precatory bean) was identified on 7/15 farms, whilst *Manihot esculenta* (Cassava) and *Crotalaria retusa* (Shak Shak, Rattleweed, Devil bean) were identified on 5/17 farms. *Nerium oleander* (Oleander), *Datura stramonium* (Prickly burr, Fire blister bush, Jimsonweed), and *Nicotiana tabacum* (Tobacco plant) were identified on 4/15 farms.

**Figure 1:**
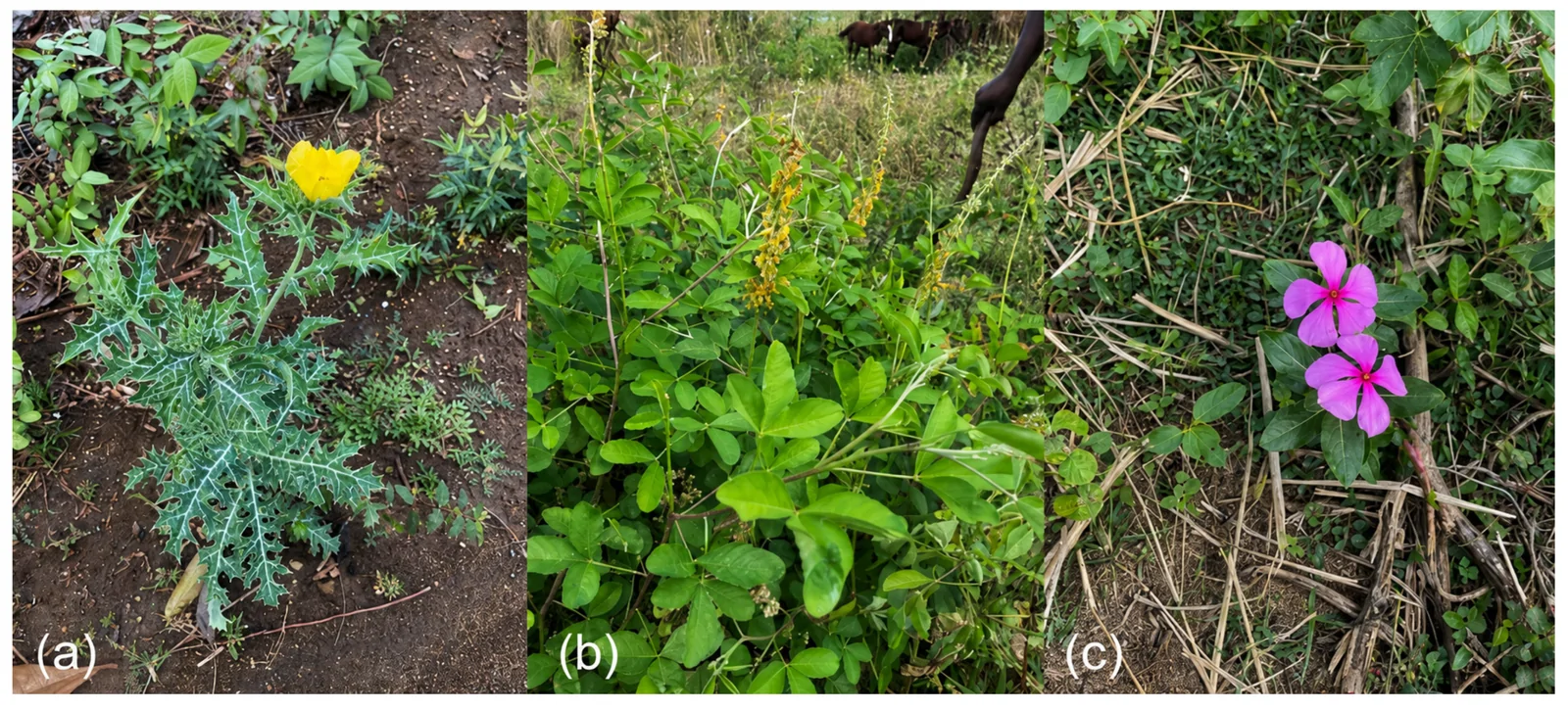
(**a**) *Argemone mexicana*, (**b**) *Crotalaria retusa*, (**c**) *Catharanthus roseus*.

Fourteen out of the 46 plants included in this study contain a variety of alkaloids as main potent phytotoxin, namely *Piper amalgo* (Candle bush, Amalgo pepper), *Solanum bahamense* (Cankerberry, Bahama nightshade), *Hymenocallis tubiflora* (Jumbee Lilly, Jumbee onion, Tuberose), *Hippeastrum puniceum* (Easter Lilly, Barbados lily), *Colubrina elliptica* (Mawbee, Soldierwood, Nakedwood), *Catharanthus roseus* (Periwinkle), *Datura stramonium* (Prickly burr, Fire blister bush, Jimsonweed), *Crotalaria retusa* (Shak Shak, Rattleweed, Devil bean), *Mimosa pudica* (Shameful, Shame lady, Sleep lady, Sensitive plant, Touch-Me-Not), *Citrus aurantium* (Sour orange, Bitter orange), *Argemone mexicana* (Mexican thistle, Mexican poppy) (Figure 1a), *Nicotiana tabacum* (Tobacco plant), *Tecoma stans* (Yellow elder, Yellow flower tree, Yellow trumpetbush, Yellow bells), and *Cuscuta americana* (Love vine, Yellow dad, American dodder).

These surveyed plant species contain biologically active alkaloids belonging to distinct structural classes. *Piper amalago* contains a diverse array of amide alkaloids bearing isobutyl, pyrrolidine, dihydropyridone, piperidine, and benzylamine moieties [36]. *Solanum bahamense* is characterized by the presence of steroidal glycoalkaloids, primarily solamargine and solasonine, which are typical constituents of the genus Solanum [37]. *Hymenocallis tubiflora* contains several *Amaryllidaceae* alkaloids, including lycorine, galanthamine, and pyrrolophenanthridine-type alkaloids [38]. Similarly, galanthamine-type alkaloids represent the principal bioactive constituents of *Hippeastrum puniceum* [39]. In contrast, species containing bisbenzylisoquinoline alkaloids are characterized by the presence of structurally complex dimeric isoquinoline alkaloids with diverse pharmacological activities. *Catharanthus roseus* contains terpenoid indole alkaloids, a diverse group of compounds with recognized pharmacological activity [40]. *Datura stramonium* is characterized by tropane alkaloids, including atropine, hyoscyamine, and scopolamine, which exert potent anticholinergic effects [41]. *Crotalaria retusa* contains pyrrolizidine alkaloids, with monocrotaline being the predominant constituent and principal toxic agent [5]. The major alkaloid of *Mimosa pudica* is mimosine, a non-protein amino acid reported to possess antiproliferative and pro-apoptotic properties [42]. In *Citrus aurantium*, synephrine is the predominant alkaloid and is known for its sympathomimetic stimulant activity [43]. *Argemone mexicana* contains a variety of alkaloids, including berberine, protopine, protopine hydrochloride, sanguinarine, and dihydrosanguinarine [44]. Among these, berberine is considered a major bioactive constituent and has been associated with adverse effects such as gastrointestinal disturbances, hepatotoxicity, hypotension, and bradycardia [44]. The primary alkaloid in *Nicotiana tabacum* is nicotine, an agonist of nicotinic acetylcholine receptors that exerts marked effects on the central nervous, cardiovascular, respiratory, and gastrointestinal systems [5]. *Tecoma stans* contains tecomine as its principal alkaloid, which has been reported to possess hypoglycemic activity [45]. *Cuscuta americana* contains multiple alkaloids, of which cuscutine is regarded as the most important constituent [46].

Nine out of 46 toxic plants contained glycosides. *Manihot esculenta* (Cassava), the well-known Cassava or tapioca, *Bambusa vulgaris* (Bamboo) and *Passiflora laurifolia* (Bell apple, water lemon) contain cyanogenic glycosides, which are toxic to livestock.

*Manihot esculenta* contains both cyanogenic and non-cyanogenic glycosides. The principal cyanogenic glycosides are linamarin and lotaustralin, which pose a toxicological risk because they can be hydrolyzed to release hydrogen cyanide. In contrast, the non-cyanogenic glycosides lack significant toxic effects [47]. *Bambusa vulgaris*, particularly its young shoots, also contains cyanogenic glycosides, with taxiphyllin being the predominant cyanogenic constituent [48]. *Passiflora laurifolia* likewise contains cyanogenic glycosides, among which tetraphyllin A and gynocardin are the major identified compounds. As with other cyanogenic plants, these metabolites have the potential to release hydrogen cyanide following enzymatic hydrolysis and the disease resembles cyanide poisoning [48].

**Figure 2:**
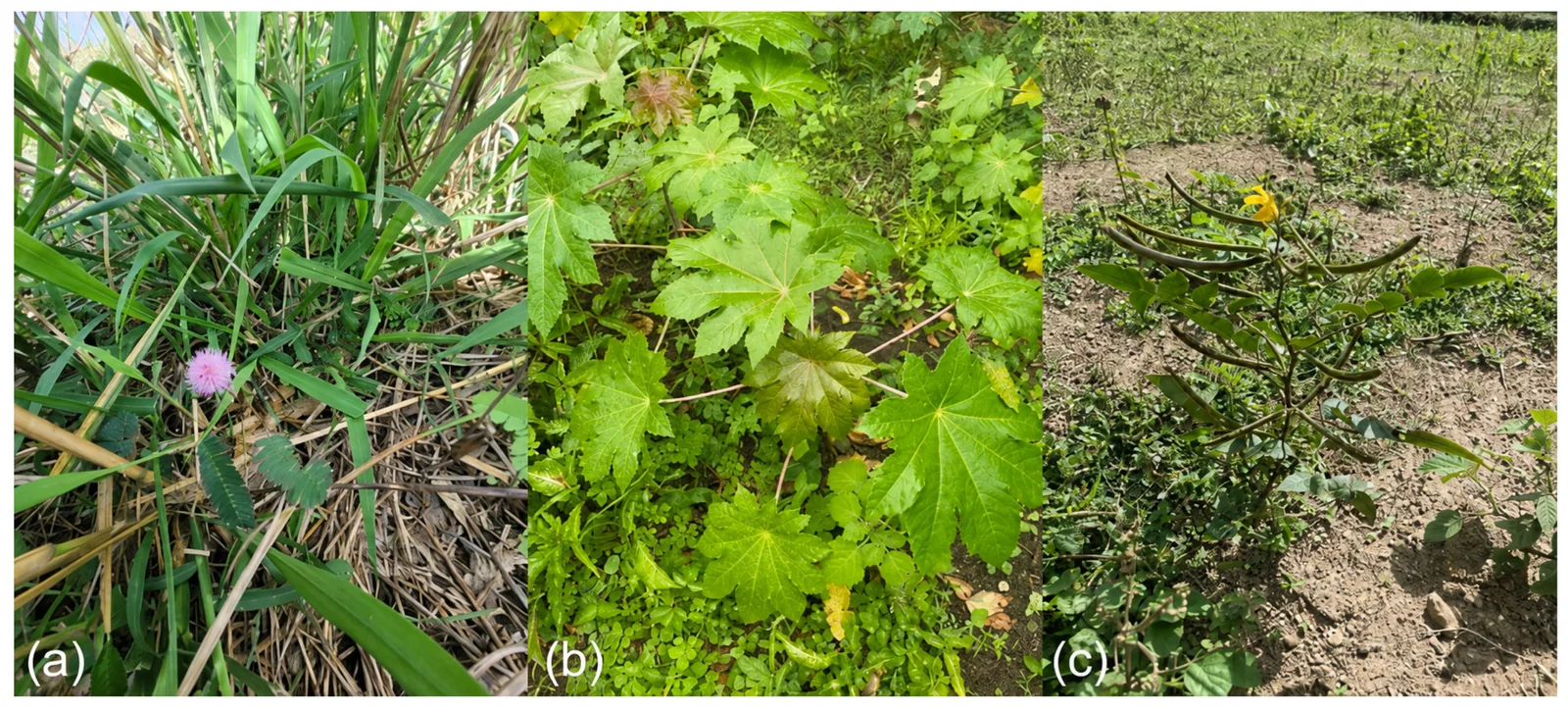
(**a**) *Mimosa pudica*, (**b**) *Ricinus communis*, (**c**) *Senna occidentalis*.

Two plants contained toxalbumins. *Abrus precatorius* (Jumbee bead, Precatory bean) and *Ricinus communis* (Castor bean) (Figure 2b) contain abrin and ricin, and ricinin, respectively. These toxalbumins inhibit cellular protein synthesis and induce severe cytotoxic effects and cell death, especially of the gastrointestinal tract mucosa, often inducing hemorrhagic gastroenteritis [5].

Thirteen out of 46 recorded plants, namely *Aloe vera* (Aloe), *Passiflora laurifolia* (Bell apple, Water lemon), *Pithecellobium unguicati* (Bread and Cheese, Cat’s claw), *Acalypha alopecuroides* (Brodah Dabbee, Foxtail copperleaf), *Chrysobalanus icaco* (Fat Pork, Cocoplum), *Caesalpinia bonduc* (Nica, Nicol, yellow nicker, Bonduc tree), *Colubrina elliptica* (Mawbee, Soldierwood, Nakedwood), *Cajanus cajan* (Pigeon pea), *Musa* paradisiaca (Plantain), *Passiflora suberosa* (Pop Bush, corkystem, passionflower), *Mimosa pudica* (Shameful, Shame lady, Sleep lady, Sensitive plant, Touch-Me-Not), *Artemisia absinthium* (Common wormwood), and *Cuscuta americana* (Love vine, Yellow dad, American dodder), contain phenolic compounds, and more specifically tannins. Those plants are common and commonly consumed by livestock; they contain variable amounts of tannins that can potentially cause diarrhea. Tannins are polyphenolic biomolecules and are broadly classified into two principal groups: condensed tannins and hydrolyzable tannins [49]. Tannins are astringent compounds that have the capacity to bind and precipitate proteins and other organic molecules such as amino acids and alkaloids. They are broadly classified into hydrolyzable and condensed tannins. Hydrolyzable tannins readily undergo hydrolysis to yield simpler compounds, with gallotannins (tannic acid) representing the most important subgroup because they release gallic acid upon degradation. Tannins constitute the principal bioactive constituents of numerous plant species and are responsible for many of their biological and toxicological properties [49].

Thirteen out of 46 plants contain either saponins or sapogenins. Those are: *Acalypha alopecuroides* (Brodah Dabbee, Foxtail copperleaf), *Solanum bahamense* (Cankerberry, Bahama nightshade), *Manihot esculenta* (Cassava), *Eryngium foetidum* (Cat Claw, Mexican culantro), *Tabeuia heterophylla* (Cedar, Stinking Cedar, Pink trumpet tree), *Lagenaria siceraria* (Gourdy Gourd, Calabash), *Abrus precatorius* (Jumbee bead, Precatory beans), *Momordica charantia* (Maiden apple, lizard food, Bitter lemon), *Achyranthes indica* (Man stronger than man, Chaff flower), *Colubrina elliptica* (Mawbee, Soldierwood, Nakedwood), *Nerium Oleander* (Oleander), *Solanum torvum* (Wild eggplant, Shushuba, Turkey berry), and *Cuscuta americana* (Love vine, Yellow dad, American dodder).

Saponins are a diverse group of glycosidic compounds widely distributed among higher plants [50]. Although they exhibit considerable structural variability, saponins share several common physicochemical and biological properties. Owing to their amphiphilic structure, which consists of a hydrophilic sugar moiety linked to a hydrophobic aglycone (genin), they possess surfactant-like characteristics that enable foam formation and the disruption of erythrocyte membranes, resulting in hemolysis [50]. Saponins exert a direct irritant effect on the gastrointestinal mucosa, resulting in a clinical syndrome that is typically mild to moderate in severity and develops within several hours following consumption [5]. Based on the chemical nature of the aglycone and associated substituents, saponins are classified into several groups, including triterpenoid, steroidal, quinone, acylated, and oligosaccharide saponins. At high concentrations, particularly steroidal saponins may exert toxic effects, primarily causing gastrointestinal irritation manifested by vomiting and diarrhea. More severe adverse effects, including hemolysis and, less commonly, hepatotoxicity, have also been reported [5,51].

Three out of 46 plants contain quinones. *Senna occidentalis* (Granny coffee, stinking weed, Coffee senna) (Figure 2c), *Senna alata* (Ringworm bush, Jumbee Shuteye, Candle bush, Ringworm bush) and *Aloe vera* (Aloe) are plants that contain quinones. *Sennaa occidentalis* that causes myopathy with skeletal muscle degeneration and necrosis (rhabdomyolysis), hepatic necrosis and acute kidney injury due to myoglobinuria [5,18,33,52]. The most prominent anthraquinones from *Aloe vera* are aloin, aloe-emodin, and barbaloin. These chemicals cause irritation of the gastrointestinal mucosa, digestive disturbance with abdominal pain and severe diarrhea. The severe purgative effect is due to an increase in mucous secretion and water content of the colon [5]. *Senna occidentalis* also contains anthraquinones like rhein, emodin and aloe-emodin [5].

Oxalic acid and it is calcium salt, calcium oxalate, are found in three out 46 plants seen, namely *Tragia volubilis* (Stinging nettle, Fireman), *Citrus aurantium* (Sour orange, Bitter orange), *Manihot esculenta* (Cassava) [53]. Other compounds with toxicity potential include steroids, ergotamine, and rotenone. *Piper amalgo* (Candle bush, Amalgo pepper) contains steroids. Ergotamine is an adrenergic agonist and is found in *Ipomoea pescaprae* (Sea-side vine, Railroad wine) [5]. *Tephrosis cirenea* (Wild Pinta, Ashen hoary pea) contains rotenone.

## 3. Discussion

Systematic documentation of toxic plant species and their geographical distribution is critical for identifying the risk of livestock toxicosis.

In the present study, the toxic plant species were classified according to their active compound. Another classification system, previously applied by Riet-Correa et al., categorizes toxic plants based on organs/systems affected, clinical signs and pathologic findings [18].

However, only 10 out of 46 plants recorded in the present survey were documented in the literature as causing clinical signs and/or pathologic changes in livestock (Table 2). The remaining 36 plant species were investigated as they contain phytochemicals with toxic potential, although their actual toxicity depends on the concentration of these compounds and the amount ingested. The recorded plants were classified into eight groups based on the nature of their main active ingredient. The plants were included in the following phytochemical groups: alkaloids, toxalbumins, glycosides, phenolic compounds, saponins and sapogenins, quinones, oxalic acid and calcium oxalates, and a miscellaneous category comprising toxic compounds not fitting the other classifications.

Alkaloids are widely distributed among plants and represent a chemically diverse group; nevertheless, only a limited number are well characterized regarding their toxicity, and not all alkaloids pose a risk to livestock [54,55,56]. Among the 14 plants recorded to contain alkaloids in this study, only five have been reported to cause disease in domestic animals.

*Crotalaria retusa* (Rattleweed) (Figure 1b), which contains pyrrolizidine alkaloids, causes hepatotoxicity and chronic progressive liver disease [5,13,14,15,18]. Pyrolizidine alkaloids themselves are biologically and toxicologically inactive and require metabolic activation. Pyrrolizidine alkaloids are metabolized in the liver to reactive pyrroles. Pyrroles alkylate DNA and disrupt RNA and protein synthesis. This interferes with mitosis, resulting in hepatocellular megalocytosis characterized by enlarged cells with polyploid nuclei [5,13,14,15,18].

*Catharanthus roseus* (Periwinkle) (Figure 1c) contains vinca alkaloids, and specifically vincristine. Vinca alkaloids act as antimicrotubule agents that block mitosis by arresting cells in the metaphase. Vincristine is commonly used as a chemotherapeutic factor for neoplasia treatment for its antimitotic properties [5,10,57]. Toxicity by ingestion of *Catharanthus roseus* causes gastrointestinal hemorrhage, disseminated intravascular coagulation, hepatic necrosis and acute kidney injury [5,10].

Anticholinergic alkaloids such as atropine, hyoscine, and hyoscyamine are found in *Datura stramonium* (Prickly burr, Fire blister bush, Jimsonweed) and act by blocking acetylcholine-mediated neurotransmission, producing clinical signs including xerostomia, tachycardia, colic, mydriasis, and visual impairment [5,11,12,18,58]. *Nicotiana Tabacum* (Tobacco plant) and *Mimosa pudica* (Shameful, Shame lady, Sleep lady, Sensitive plant, Touch-Me-Not) (Figure 2a) contain the pyridine alkaloids nicotine and mimosine, respectively. Nicotine acts as an agonist at nicotinic acetylcholine receptors (nAChRs), which are found throughout the nervous system, including the central nervous system, autonomic nervous system, and neuromuscular junctions. At low doses, nicotine stimulates nAChRs, but causes receptor desensitization and neuromuscular blockade at higher exposures, with cardiovascular and respiratory toxicity reported, including fatal intoxication in ruminants [17,59]. Mimosine toxicity primarily stems from its ability to inhibit DNA synthesis by interfering with ribonucleotide reductase, a crucial enzyme for DNA replication, and by chelating iron, a cofactor for this enzyme. Ingestion of *Mimosa pudica* by horses and other equines can cause hair loss (alopecia), lethargy, decreased appetite, and excessive salivation [5,16].

Glycosides are widely present in plants, such as cyanogenic glycosides, where hydrogen cyanide (HCN) is released following enzymatic hydrolysis. HCN disrupts cellular respiration by blocking the electron transport chain through interaction between cyanide ions and ferric iron in the cytochrome oxidase system [60]. Clinical signs include vomiting, diarrhea, depression and stupor [60]. Particularly, cassava leaf ingestion and toxicosis lead to neuronal, thyroid follicular cell and hepatic cell vacuolation [5,8,9,18,61].

Cardiac glycosides inhibit the Na^+^/K^−^ pump, leading to increased intracellular calcium and potentially fatal arrhythmias [62]. *Nerium oleander* contains oleandrin, a cardiac glycoside (cardenolide), which causes endo- and myocarditis and renal tubular degeneration; associated gastrointestinal hemorrhage is likely attributable to the plant’s terpenoid saponins [5,35,61,63,64].

Toxalbumins express their toxic action by inhibiting protein synthesis [5,34]. *Abrus precatorius* contains abrin, a highly toxic lectin (toxalbumin) that induces severe hemorrhagic gastroenteritis, vomiting and neurological signs, including seizures [5,34]. Similarly, *Ricinus communis* (Figure 2b), which contains ricin (toxalbumin) and ricinine (alkaloid), causes hemorrhagic gastroenteritis, myocarditis, hepatic cell necrosis and acute kidney injury [5,30,31,32,65].

Phenolic compounds are widely present in plants and are considered beneficial to mammalian health via their antioxidant properties; however, toxic effects have also been reported [66]. Phenolic compounds are organic molecules characterized by the presence of one or more hydroxyl groups attached to an aromatic ring. This structural feature confers polarity and enables the formation of hydrogen bonds, properties that contribute significantly to their chemical reactivity and biological activities. Phenolic compounds are generally classified as simple phenols, which contain a single aromatic ring with one or more hydroxyl substituents, and polyphenols, which possess multiple phenolic units. Major classes of polyphenols include flavonoids, lignans, tannins, and phenolic acids [67]. Among these compounds, tannins are the phenolic constituents most frequently implicated in cases of animal poisoning. Mechanisms of toxicity of phenolic compounds include: cellular injury, coagulative necrosis, mitochondrial dysfunction, and DNA damage, and downregulation of adhesion molecules [66,68].

Saponins and sapogenins may induce hemolysis, mucous membrane irritation, cell death, abortion and intestinal epithelial cell lysis.

Saponins are naturally occurring glycosides that exhibit soap-like properties when dispersed in water. Structurally, they consist of a hydrophobic aglycone (sapogenin) linked to one or more hydrophilic sugar moieties [51]. Based on the chemical nature of the aglycone and associated substituents, saponins are classified into several groups, including triterpenoid, steroidal, quinone, acylated, and oligosaccharide saponins. At high concentrations, particularly steroidal saponins may exert toxic effects, primarily causing gastrointestinal irritation manifested by vomiting and diarrhea. More severe adverse effects, including hemolysis and, less commonly, hepatotoxicity, have also been reported [5,51,69].

Quinones possess strong oxidative properties and are cytotoxic, immunotoxic and carcinogenic [70].

Oxalic acid and calcium oxalates were identified as the active ingredients in three plant species. Excess ingestion of those chemical compounds can lead to oxalate crystal deposition within the urinary system, which can lead to acute renal tubular degeneration and subsequently renal failure [53].

Additional toxic compounds identified include steroids, ergotamine, and rotenone. Ergotamine is an adrenergic agonist [5]. Rotenone inhibits mitochondrial complex I, resulting in impaired ATP production, oxidative stress and eventually cell death. Rotenone toxicity may lead to vomiting, nausea and neurodegenerative disease [71].

Although many of the identified plants have a toxic potential, the mere presence of a toxic plant in a pasture does not necessarily indicate that intoxication has occurred. The likelihood of poisoning depends on several factors, including the plant species involved, the concentration of toxic constituents, the quantity consumed, the duration of exposure, and animal susceptibility. For example, plants containing pyrrolizidine alkaloids, such as *Crotalaria retusa*, typically require prolonged exposure before clinical disease develops. Feed contaminated with as little as 0.05% *C. retusa* seeds has been reported to cause intoxication in chicks when consumed over several weeks [5]. In contrast, *Nerium oleander* is highly toxic, and ingestion of approximately 50 mg/kg body weight may be fatal to horses within 2 to 3 days [72]. Plants containing cyanogenic glycosides, such as *Manihot esculenta*, may produce peracute to acute toxicosis, with clinical signs developing within minutes of ingestion and rapidly progressing in severity [5]. Similarly, *Ricinus communis* is toxic to all animal species, with clinical signs typically appearing within 24 h after consumption. Reported toxic doses are approximately 0.1 g/kg in horses, 1.25 g/kg in sheep, 1.4 g/kg in pigs, 2 g/kg in cattle, and 5.5 g/kg in goats [73]. The toxicity of *Senna occidentalis* is associated with myotoxins that induce skeletal muscle degeneration and cardiomyopathy following the ingestion of substantial quantities of plant material [5]. However, outbreaks are generally sporadic and usually affect only a limited number of animals at a time. Ingestion of *Abrus precatorius* seeds causes an acute toxicosis to which all livestock species are susceptible. Reported lethal doses are approximately 100 mg/kg body weight in horses, 600 mg/kg in cattle, and more than 2 g/kg in goats [5]. Poisoning by *Catharanthus roseus* generally requires prolonged ingestion of foliage. Clinical manifestations include the abrupt onset of ataxia, lateral flexion of the neck, tremors, and seizures, followed by coma and death within 1 to 2 days [5]. Although *Datura stramonium* contains potent tropane alkaloids, intoxication is relatively uncommon because the plant is unpalatable. Toxicity is greatest in mature plants and persists after desiccation. Consequently, dried plant material contaminating hay may represent a significant source of exposure. Administration of leaves or fruits at approximately 1% body weight per day to sheep and goats has been reported to induce clinical signs after two or more days of consumption; however, lethal intoxication generally requires longer-term exposure [5]. In contrast, poisonings caused by *Mimosa pudica* are exceedingly rare, despite the presence of potentially toxic constituents such as mimosine and saponins, making intoxication in livestock unlikely under natural conditions [5]. *Nicotiana tabacum* contains multiple alkaloids, with nicotine occurring at the highest concentrations. Because alkaloid content varies considerably among plant populations and environmental conditions, toxic dose estimates are difficult to establish. Experimental studies have shown that ingestion of dry plant material equivalent to 0.07% body weight or fresh plant material equivalent to 0.75% body weight can induce clinical signs in cattle, sometimes within minutes of consumption. Ingestion of fresh plant material exceeding 2% body weight may result in death [5]. Therefore, while the identification of toxic plant species in a pasture highlights a potential risk factor, it should not be interpreted as definitive evidence of poisoning without consideration of exposure level, duration of ingestion, and supporting clinical or epidemiological findings.

Assessment of plant distribution between highland and lowland farms indicated that many species were present in both environments, although some showed geographic preference (Table 1). A major limitation of this analysis was the small and unequal number of farms surveyed. The farms visited were selected from the registered farms in the data by the student’s ruminant club at Ross University School of Veterinary Medicine and by local connections of one of the authors. An effort was made to include farms of a variety of geographical terrains, specifically highlands and lowlands. It is acknowledged by the authors that the type of livestock on the farms is not representative, i.e., we do not include an equal number of farms with different farmed species, and this is a limiting factor. Of the 10 plant species documented to cause livestock toxicity (*Manihot esculenta*, *Ricinus communis*, *Senna occidentalis*, *Abrus precatorius*, *Nerium oleander*, *Catharanthus roseus*, *Datura stramonium*, *Crotalaria retusa*, *Mimosa pudica*, and *Nicotiana tabacum*), six primarily contained alkaloids, two toxalbumins, three glycosides, and one contained phenolic compounds (tannin) [8,9,10,11,12,13,14,15,16,17]. Those plants are widespread on the island and present in both highland and lowland farms (Table 1).

Although most of those plants are unpalatable, ingestion may occur during periods of forage scarcity, and dry weather, particularly during the Caribbean dry season (December to May) or when plants are inadvertently harvested and fed to livestock. Limitations to this study include the reliance on limited regional literature and a restricted number of surveyed farms. Another limitation is that *Pteridium* spp. (fern) and *Lantana camara* (Wild sage, Tickberry, Shrub verbena, Spanish flag), two well-known poisonous plants, were not included in the study.

Farmers expressed great interest in improving livestock management and were particularly interested in knowing the prevalence of toxic plants on pastures. In cases of livestock losses, parasitism was commonly suspected, although plant toxicosis could not be excluded. Notably, when filling out the questionnaire, some farmers acknowledged feeding plants known to be potentially toxic.

Comparatively, few peer-reviewed studies have documented toxic plants affecting livestock in the Caribbean. In one review paper, the poisonous plants of South America, which should be similar to the ones in the Caribbean region, are reported [18]. In comparison, almost half of the poisonous plants present in our paper are recorded in South America. Numerous studies describing plant-related poisonings have been published in South America. *Crotalaria* spp. are a frequent cause of poisoning in horses and cattle in Brazil [19,20]. A survey study of ranchers carried out in Brazil by Paim et al. in 2023 identified numerous plants causing losses in cattle [74]. In a 2023 study, Riet-Correia et al. reviewed 219 plants involved in poisoning episodes in livestock in South America and classified them according to the main organ system affected [18].

## 4. Conclusions

In conclusion, plant species toxic to livestock are present on the island of St. Kitts and represent a potential threat to animal health, particularly to livestock grazing in fields/pastures or when animals are being fed with those toxic plants.

The true frequency of plant toxicosis remains unknown, largely due to underreporting by farmers, the absence of systematic surveillance, and limited awareness among both producers and animal health professionals.

The implementation of improved surveillance systems, combined with targeted education of farmers and veterinary professionals, would facilitate early diagnosis of toxicoses, clarify the scale of the problem, and help determine which plant species are most commonly involved and which livestock populations are most commonly affected. Documentation of the geographic distribution of toxic plants may further assist in identifying high-risk areas.

To our knowledge, this study represents the first systematic documentation of plant species toxic to livestock on St. Kitts. Further investigations are warranted to evaluate the distribution of toxic plants across other Caribbean islands and to explore associations between plant prevalence and livestock poisoning outbreaks within the region.

## 5. Materials and Methods

This is a prospective, descriptive study conducted over a one-year period (September 2023 to September 2024) on 17 farms from all parishes of Saint Kitts.

The first step of the research methodology was to identify any relevant literature describing the flora of St. Kitts and the identification of potentially toxic plants to livestock. To accomplish this objective, an extensive bibliographic study was performed to identify any peer-reviewed, published paper or book related to plants potentially toxic to livestock on the island of Saint Christopher (St. Kitts). We recovered a report written by Horwith, B., K. Lindsay, and B. Potter, titled “A biodiversity profile of St. Kitts and Nevis” [75], and 2 books by Dr Whittaker and Dr Morris titled “Plants around us, Part 1 & 2 [6,7]. These books describe a large number of plants used for their medicinal and nutritional properties as well as ornamentally in St. Kitts and Nevis. From this long list of plants, we selected 50 (reduced to 46 after further review) with potential toxic effects if ingested by livestock and created a questionnaire addressed to local farmers, which was completed by one of the authors of this paper by interviewing local farmers, examining the farm and pastures, recording toxic plants on the farm and assessing whether livestock has access to or grazes on the toxic plants. The 4 plants excluded from the study after further review, namely *Gossypium barbadense*, *Eleutherine bulbosa*, *Allium cepa*, and *Microtea debilis,* are either non-toxic (*Eleutherine bulbosa* and *Microtea debilis)* or a high dose was required to cause toxicity, and this was not applicable on farms of St. Kitts. Other questions included in the questionnaire were related to the type of livestock on the farm, grazing and feeding policies, and whether any additional feed supplements are used. We visited 17 farms, on highlands and lowlands, representative of all the parishes of the island.

The inclusion criteria encompassed livestock farmers from all parishes of the island who expressed a willingness to participate in the study. Farms and pastures located across a range of altitudes were included to account for the influence of elevation on plant species distribution. A convenience sampling approach was employed for participant selection. The total livestock population could not be accurately quantified because farm records were frequently incomplete, inconsistently maintained, or unavailable. Furthermore, livestock were predominantly managed under free-range systems, and animal numbers reported by farmers generally represented approximate estimates rather than precise counts.

**Figure 3:**
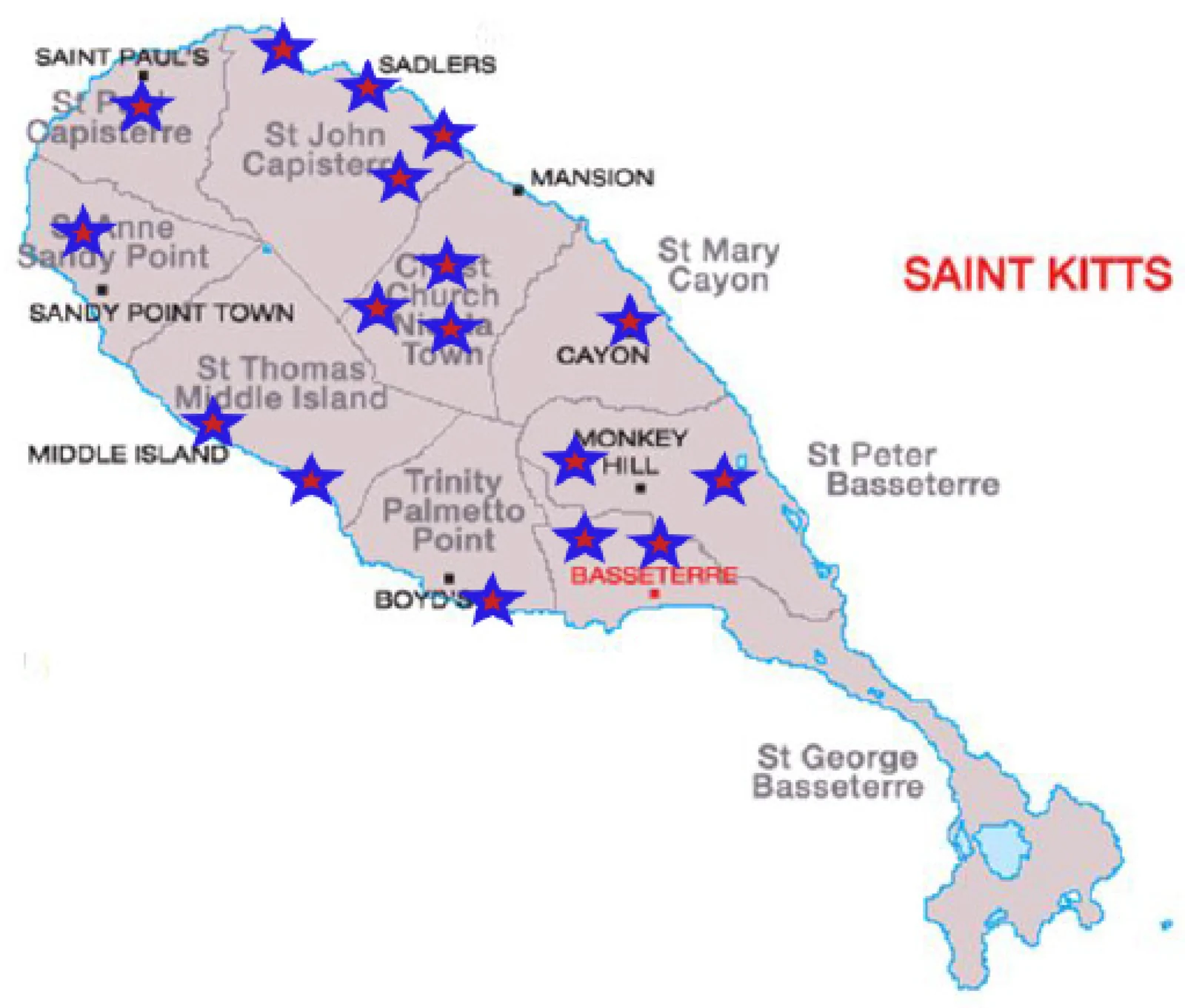
Map of St. Kitts. Locations of farms visited and surveyed during the study are marked with a star.

The geographic position of the visited farms and the altitude at which these are located (lowland/highland) were recorded (Table 1 and Figure 3).

The geographic coordinates of all participating farms were recorded using GPS and subsequently used to generate the map illustrating farm locations included in the study (Figure 3). A semi-quantitative assessment of plant abundance was conducted at each site, and the common and dominant plant species identified within each farm or pasture were documented and reported.

## Figures and Tables

**Table 1:** Plants, toxins, part of plant where toxins are found, and farms where the plants were recorded.

Plant Name (Indigenous and Scientific)	Plant Secondary Metabolites	Part of Plant Where Toxin Is Present	Species Affected	Number and Name of Farms Present	References
Aloes (*Aloe vera*)	Aloin (anthraquinone glycoside), Anthraquinones	Leaf, fruit	All livestock	9 (Lodge village L, Molineux L, Dieppe H, Sadwell (Basseterre) H, Brumaire L, West Commodore H, Fig tree H, Cedar grove H, St Peters H)	[6,21]
2. Bamboo (*Bambusa vulgaris*)	Cyanogenic glycosides	Shoot	Horses, rarely cattle	2 (West Commodore H, Cedar Grove H)	[5,6]
3. Bell Apple (*Passiflora laurifolia*)	1. Tannins (phenolic compounds) 2. Cyanogenic glycosides	1. Young fruit, seed 2. Leaf, stem, unripe fruit	Humans, No specific mention of toxicity to grazing animals	3 (Lodge village L, Molineux L, Cedar Grove H)	[6,22]
4. Breadfruit (*Articarpus altilis*)	Tannins (phenolic compound)	Leaf	No report of toxicity in livestock	10 (Lodge village L, Molineux L, Tabernacle L, Dieppe H, Cayon L, Sadwell (Basseterre) H, Brumaire L, West Commodore H, Fig tree H, Cedar Grove H)	[6,23]
5. Bread and cheese (*Pithecellobium unguicati*)	Tannins (phenolic compounds)	Bark	No reports in animals	4 (Lodge village L, Dieppe H, Boyds H, Wingfield manor (Old Road) H)	[6]
6. Brodah dabbee (*Acalypha allpecuroides*)	Saponins, tannins (phenolic compounds), polyphenols	All parts	All livestock—low risk	4 (Lodge village L, Dieppe H, Boyds H, West Commodore H)	[5,6]
7. Candle bush (*Piper amalgo*)	Triterpenes, steroids, alkaloids	All parts	No reports in animals	5 (Lodge Village L, Molineux L, Tabernacle L, Bellevue L, Middle Island H)	[6]
8. Cankerberry (*Solanum bahamense*)	Steroidal alkaloids, sapogenins, sesquiterpenes	All parts	All livestock	7 (Lodge village L, Tabernacle L, Bellevue L, Dieppe H, Cayon L, Boyds H, RUSVM L)	[5,6]
9. Cassava (*Manihot esculenta*)	1. Cyanogenetic glycosides, 2. Saponins, oxalic acid	1. All parts 2. Tuber	All livestock	5 (Lodge village L, Phillips village H, Cayon L, Fig tree H, Cedar Grove H)	[5,6]
10. Castor bean (*Ricinus communis*)	Ricin (Lectin)	Seed	All livestock	15 (Lodge village L, Molineux L, Tabernacle L, Bellevue L, Dieppe H, Cayon L, Boyds H, Brumaire L, West Commodore H, Middle Island H, Fig tree H, Wingfield manor (Old Road) H, Cedar Grove H, St Peters H, RUSVM L)	[5,6]
11. Cat Claw (*Eryngium foetidum*)	Saponins	Root	No reports in livestock species	5 (Bellevue L, West Commodore H, Fig tree H, Cedar Grove H, St Peters H)	[6]
12. Cedar, Stinking Cedar (*Tabebuia heterophylla*)	Saponins	Bark	No reports in livestock species	3 (Bellevue L, Middle Island H, Wingfield manor (Old road) H)	[6,24]
13. Fat pork (*Chrysobalanus icaco*)	Tannins (phenolic compounds)	All parts	No reports in livestock species	4 (Lodge village L, St Peters H, Fig Tree H, Cedar Grove H)	[6]
14. Gourdy Gourd (*Lagenaria siceraria*)	Steroidal sapogenins	Leaf	Humans, No reports in livestock species	1 (Fig Tree H)	[6,25]
15. Granny coffee, stinking weed (*Senna occidentalis*)	Oxymethyl anthraquinones	All parts	Horses, rabbits, chicken, pigs, cattle	10 (Lodge village L, Molineux L, Tabernacle L, Dieppe H, middle island H, Fig tree H, Wingfield manor (Old Road) H, Cedar Grove H, St Peters H, RUSVM L)	[5,6]
16. Jumbee bead (*Abrus precatorius*)	1. Abrin (Lectin), 2. Sweet glycosides, 3. Saponins	1. Seed, 2. Leaf, 3. All parts	All livestock	7 (Lodge village L, Bellevue L, Dieppe H, Sadwell (Basseterre) H, Middle Island H, Fig Tree H, Cedar Grove H)	[5,6]
17. Jumbee onion, Jumbee lilly (*Hymenocallis tubiflora*)	Alkaloids	Rhizome, bulb, leaf	No reports in livestock species	2 (St Peters H, Cedar Grove H)	[6]
18. Barbados lily, Easter lily (*Hippeastrum puniceum*)	Alkaloids, Lycorine (alkaloid)	Bulb	Dog, No reports in livestock species	1 (Brumaire L)	[6,26]
19. Maiden apple, Lizard food (*Momordica charantia*)	Diosgenin (sapogenin)	Fruit	No reports in livestock species	13 (Lodge village L, Molineux L, Tabernacle L, Bellevue L, Dieppe H, Cayon L, Boyds H, Sadwell (Basseterre) H, Brumaire L, West Commodore H, Fig Tree H, Cedar Grove H, St Peters H)	[6]
20. Man stronger than man (*Achyranthes indica*)	Saponins	Seed	No reports in livestock species	8 (Lodge village L, Bellevue L, Dieppe H, Boyds H, Brumaire L, Fig Tree H, Cedar Grove H, St Peters H)	[6]
21. Mawbee (*Colubrina elliptica*)	Saponins, tannins (phenolic compound), alkaloids	Bark	No reports in livestock species	1 (Fig Tree H)	[6]
22. Black mustard (*Brassica nigra*)	Sinigrin (glycoside)	Seed	Cattle, sheep, goats	7 (Bellevue L, Boyds H, Middle Island H, Fig Tree H, Wingfield manor (Old Road) H, Cedar Grove H, St Peters H)	[5,6]
23. Nica, Nicol, Yellow nicker (*Caesalpinia bonduc*)	Tannins (phenolic compounds)	Green seed	No reports in livestock species	7 (Lodge village L, Molineux L, Tabernacle L, Bellevue L, Brumaire L, Fig Tree H, St Peters H)	[6]
24. Oleander (*Nerium oleander*)	Oleandrin and other glycosides, triterpenoid saponins	All parts	All livestock	4 (Lodge village L, Brumaire L, Fig Tree H, St Peters H)	[5,6]
25. Periwinkle (*Catharanthus roseus*)	Vincristine (vinca alkaloid), alkaloids	All parts	Cattle, sheep, horse	11 (Lodge village L, Molineux L, Tabernacle L, Bellevue L, Dieppe H, Boyds H, Shadwell (Basseterre) H, middle island H, Fig tree H, Cedar Grove H, St Peters H)	[5,6,10]
26. Pigeon pea (*Cajanus cajan*)	Tannins (phenolic compounds)	Leaf, root	No reports in livestock species	6 (Lodge village L, Tabernacle L, West Commodore H, Fig Tree H, Cedar Grove H, St Peters H)	[6]
27. Plantain (*Musa paradisiaca*)	Tannins (phenolic compound)	All parts	No reports in livestock species	14 (Lodge village L, Molineux L, Tabernacle L, Bellevue L, Phillips village H, Dieppe H, Boyds H, Shadwell (Basseterre) H, Brumaire L, West Commodore H, Fig Tree H, Wingfield manor (Old Road) H, Cedar Grove H, St Peters H)	[6]
28. Pop bush (*Passiflora suberosa*)	Tannins (phenolic compounds)	All parts	No reports in livestock species	4 (Lodge village L, Bellevue L, Boyds H, Fig Tree H)	[6]
29. Prickly burr, Fire blister bush (*Datura stramonium*)	Alkaloids: atropine, hyoscine, hyoscyamine (anticholinergic)	Leaf, seed	Cattle, goats, horses, sheep, swine, and poultry	4 (Bellevue L, Boyds H, Fig Tree H, St Peters H)	[6,27]
30. Rabbit food (*Leonotis nepetifolia*)	Resins (often contain terpenes)	Leaf, flowers	No reports in livestock species	4 (Molineux L, Tabernacle L, Fig Tree H, Wingfield manor (Old Road) H)	[6]
31. Ringworm bush, Jumbee shuteye (*Senna alata*)	Emodin (anthraquinone)	All parts	All livestock	6 (Bellevue L, Brumaire L, West Commodore H, Middle Island H, Fig Tree H, Wingfield manor (Old Road) H)	[5,6]
32. Roucou, Knuckle seed (*Bixa orellana*)	Sesquiterpenes	Leaf	No reports in livestock species	4 (Brumaire L, West Commodore H, Cedar Grove H, St Peters H)	[6]
33. Sage (*Cordia curassavica*)	Terpineol (terpenoid)	Leaf	No reports in livestock species	9 (Lodge village L, Tabernacle L, Dieppe H, Shadwell (Basseterre) H, middle island H, Fig Tree H, Wingfield manor (Old Road) H, Cedar Grove H, St Peters H)	[6]
34. Sea side vine (*Ipomoea pes-caprae*)	Ergotamine (adrenergic agonist)	All parts	Goats, sheep, cattle, buffalo	2 (Fig Tree H, Cedar Grove H)	[5,6]
35. Shak Shak (*Crotalaria retusa*)	Pyrrolizidine alkaloids	All parts	All livestock, mainly horses	5 (Bellevue L, Boyds H, Shadwell (Basseterre) H, Fig tree H, Wingfield manor (Old Road) H)	[5,6]
36. Shameful, Shame lady, Sleep lady (*Mimosa pudica*)	Alkaloids, Tannins (phenolic compounds)	All parts	Aqueous and methanolic extract toxic to goat, sheep and cattle	13 (Molineux L, Tabernacle L, Bellevue L, Phillips village H, Dieppe H, Boyds H, Shadwell (Basseterre) H, West Commodore H, Middle Island H, Fig Tree H, Wingfield manor (Old Road) H, Cedar Grove H, St Peters H)	[6,28]
37. Sour orange (*Citrus aurantium*)	1. Oxalic acid, 2. Alkaloids	1. Fruit, 2. Mature leaf	No reports in livestock species	5 (Lodge village L, Molineux L, Bellevue L, Fig Tree H, Wingfield manor (Old Road) H)	[6]
38. Stinging Nettle (*Tragia volubilis*)	Calcium oxalate	All parts	All livestock	15 (Lodge village L, Molineux L, Tabernacle L, Bellevue L, Phillips village H, Dieppe H, Boyds H, Brumaire L, West Commodore H, Middle Island H, Fig Tree H, Wingfield manor (Old road) H, Cedar Grove H, St Peters H	[5,6]
39. Mexican thistle (*Argemone mexicana*)	Berberine (alkaloid)	Leaf	All livestock	14 (Molineux L, Tabernacle L, Bellevue L, Dieppe H, Cayon L, Boyds H, Shadwell (Basseterre) H, Brumaire L, Middle Island H, Fig Tree H, Wingfield manor (Old road) H, Cedar Grove H, St Peters H, RUSVM L)	[5,6]
40. Tobacco (*Nicotiana tabacum*)	Nicotine, other alkaloids	Leaf	All livestock	4 (Molineux L, Tabernacle L, Bellevue L, Fig Tree H)	[5]
41. Wild eggplant, Shushuba (*Solanum torvum*)	Sapogenins	All parts	Cattle, sheep, horses, goats	10 (Lodge village L, Molineux L, Tabernacle L, Bellevue L, Cayon L, Boyds H, West Commodore H, Middle Island H, Fig Tree H, Cedar Grove H)	[5]
42. Wild pinta (*Tephrosis cinerea*)	Rotenone (rotenoid)	All parts	Potentially toxic to all livestock	2 (Boyds H, Fig Tree H)	[6,29]
43. Wormwood (*Artemisia absinthium*)	Tannins (phenolic compounds), hydroxycoumarins	All parts	Horse, sheep, goats, cattle	7 (Lodge village L, Molineux L, Tabernacle L, Boyds H, Middle Island H, Fig Tree H, Cedar Grove H)	[5,6]
44. Worry wine, Verry wine (*Stachypherta jamaicanensis*)	Glycoside compounds	All parts	No reports in livestock species	8 (Lodge village L, Tabernacle L, Dieppe H, Boyds H, Brumaire L, West Commodore H, Fig Tree H, St Peters H)	[6]
45. Yellow elder, Yellow flower tree (*Tecoma stans*)	1. Alkaloids, 2. Resins	1. Leaf, wood, seed, 2. All parts	No reports in livestock species	2 (Middle Island H, Fig Tree H)	[6]
46. Yellow dad, dodder, love vine (*Cuscuta americana*)	Glycosides, tannins (phenolic compounds), saponins, quercetin (polyphenol), alkaloids	All parts	Cattle, horses, rabbits, potentially other livestock species	8 (Tabernacle L, Boyds H, Shadwell (Basseterre) H, Brumaire L, Wingfield manor (Old Road) H, Cedar Grove H, St Peters H, RUSVM L)	[5,6]

Legend: H. Highland, L. Lowland, RUSVM: Ross University of Veterinary Medicine.

**Table 2:** Plants recorded to be toxic to livestock in the literature.

No.	Plant Name Common (Scientific)	Plant Secondary Metabolites	Species Affected	Clinical Signs/Pathology (Macroscopic and Microscopic Findings)	References
1	Cassava (*Manihot esculenta*)	Cyanogenic glycosides	All livestock	CNS (Central Nervous System (excitement), Thyroid, Liver cell vacuolation	[5,6]
2	Castor bean (*Ricinus communis*)	Ricin (lectin)	All livestock	GIT (Gatrointestinal tract) (diarrhea/gastroenteritis), Cardiac hemorrhage, hepatic necrosis, acute kidney injury (AKI)	[5,6]
3	Granny coffee, Stinking weed (*Senna occidentalis*)	Anthraquinones	Horses, rabbits, chicken, pigs, cattle	Myopathy, Skeletal muscle degeneration and necrosis (rhabdomyolysis). Centrilobular hepatic necrosis. Secondary myoglobinuria and acute kidney injury	[5,6]
4	Jumbee bead (*Abrus precatorius*)	Abrin (lectin)	All livestock	Stomach ache, vomiting, GIT (hemorrhagic gastroenteritis), CNS clinical signs	[5,6]
5	Oleander (*Nerium oleander*)	Oleandrin (cardiac glycoside)	All livestock	GIT (hemorrhagic gastroenteritis), hemorrhagic endo- and myocarditis.	[5,6]
6	Periwinkle (*Catharanthus rosea*)	Vincristine (vinca alkaloid), Alkaloids (acatharanthin and others)	Cattle, sheep, horse	GIT hemorrhage, DIC (Disseminated Intravascular Coagulopathy), hepatic necrosis, kidney: acute tubular necrosis.	[5,6,10]
7	Prickly burr (*Datura stramonium*)	Alkaloids (Atropine, Hyoscine, Hyoscyamine)	Cattle, goats, horses, sheep, swine, and poul-try	Anticholinergic action. High heart rate, dilated pupils, loss of vision, dry mouth and other mucous membranes. Later nausea, loss of muscle coordination, aggressive behavior to name a few, possible death. Colic in horses	[5,6,27]
8	Shak Shak (*Crotalaria retusa*)	Pyrrolizidine alkaloids	All types of live-stock, mainly horses	Hepatotoxicity	[5,6]
9	Shamelady/Sleeping lady (*Mimosa pudica*)	Mimosine (alkaloid)	Aqueos and methanolic ex-tract toxic to goat, sheep and cattle	Horses, donkeys: hair loss, symmetric. Endocrine dermatosis. Cattle, Sheep, Goats: severe nephrotoxicity and perineal edema	[6,28]
10	Tobacco (*Nicotiana tabacum*)	Nicotine (alkaloid)	All livestock	Nervous symptoms (generally unpalatable). Animals eat in periods of dry weather/scarcity of food	[5]

## Data Availability

The original contributions presented in this study are included in the article/Supplementary Material. Further inquiries can be directed to the corresponding author.

## Supplementary Materials

The following supporting information can be downloaded at: https://www.mdpi.com/article/10.3390/toxins18090380/s1, Table S1: Farm questionnaire, Toxic plants in St. Kitts.

## Abbreviations

The following abbreviations are used in this manuscript:

ATP	Adenosine triphosphoric acid
DNA	Deoxyribonucleic acid
HCN	Hydrogen cyanide
nAChR	nicotinic acetylcholine receptors
Na^+^/K^−^ pump	Sodium/potassium pump
RNA	Ribonucleic acid
