# Supplementary material for: Toxic Plants Potentially Impacting the Health Status of Livestock in St. Kitts—A Surveillance Study
Source: Toxins (Basel). 2026 Sep 3;18(9):380. doi: 10.3390/toxins18090380 (PMC13611601; doi:10.3390/toxins18090380)
Supplement: Supplementary file 1 [file toxins-18-00380-s001.zip › toxins-4467662-supplementary.pdf]

ROSS UNIVERSITY
SCHOOL OF VETERINARY MEDICINE

Toxic Plants in St. Kitts questionnaire

Personal information

- Name in full:_______________________________________
- Location (Village name, farm name, GPS): ______________________

Farm questions

- Type of livestock: a. Cattle, b. Sheep, c. Goats, d. Pigs, e. other (please specify)_________
- Graze location: a. Fenced fields, b. Free range, c. other (please specify): ______________
- Additional feed provided: a. grains, b. pellets, c. silage, d. other (please specify):_________
- Feed supplements: _________________________

<u>Toxic Plant questions</u>

Table S1: Farm questionnaire, Toxic plants in St. Kitts.

Have you ever seen the following plants on the grazing fields:

**Plant name(s) (scientific name)**	**Species affected**	**Seen in fields**	**Animals seen grazing the plant**	**Picture**
Aloes, simpee wiry (*Aloe vera*) gel				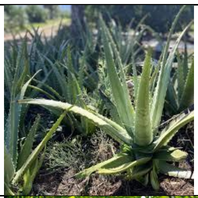
Bamboo (*Bambusa vulgaris*) shoots				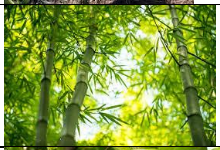
Bell apple (*Passiflora laurifolia*)				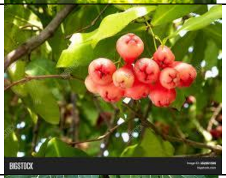
Breadfruit, canaan fruit (*Artocarpus altilis*) leaves				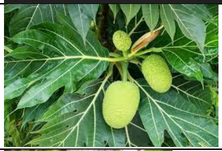
Bread and cheese (*Pithecellobium unguis-cati*) bark				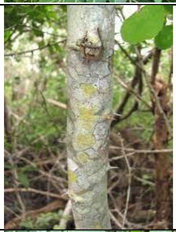
Brodah dabbee, brodah Feddie (*Acalypha Alopecuroides*)				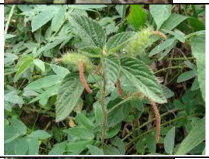
Candle bush, candle wood (*Piper amalgo*)				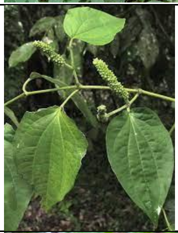
Cankerberry (*Solanum bahamense*)				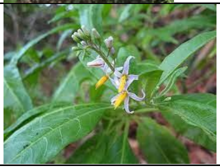

Cassava (*Manihot escelenta*) tuber				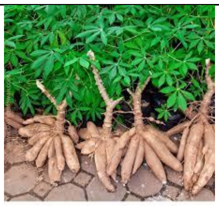
Castor bean (*Ricinus communis*)				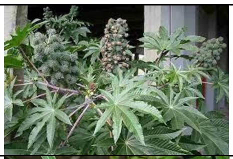
Cat-claw, fit weed, Cla-cla (*Eryngium foetidum*)				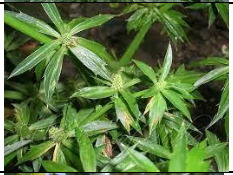
Cedar, stinkin cedar, white cedar (*Tabebuia heterophylla*)				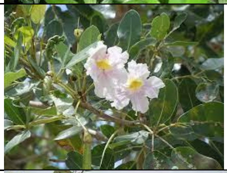
Cotton (*Gossypium barbadense*) seed				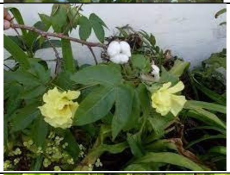
Fat pork (*Chrysobalanus icaco*)				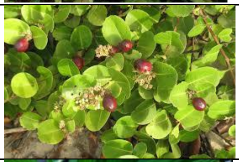
Garden maize (mace), blood root, mountain ginger (*Eleutherine bulbosa*)				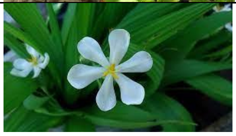
Gourdy, Gourd (*Lagenaria siceraria*) leaves				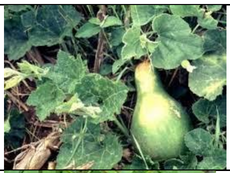
Granny coffee, stinking weed, wild coffee (*Cassia occidentalis*) root				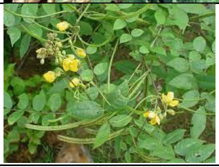

Jumbee bead (*Abrus precatorius*) seed and leaves				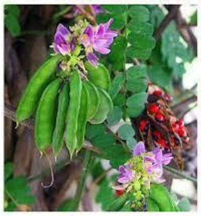
Jumbee onion, jumbee lilly (*Hymenocallis tubiflora*)				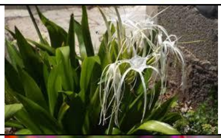
Lily, easter (*Hippeastrum puniceum*) bulb				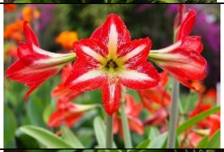
Maiden apple, maiden blush, lizard food, washer woman (*Momordica charantia*) fruit				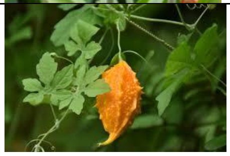
Man stronger than man, man better man, devil's horse whip (*Achyranthes indica*) seed				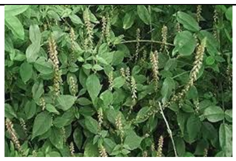
Mawbee (*Colubrina elliptica*) bark				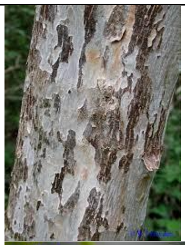 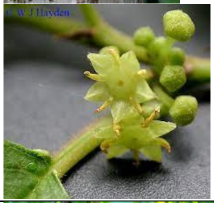
Mustud, mustard (*Brassica nigra*)				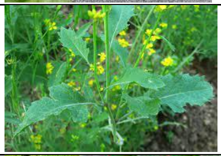
Nica, nicol, yellow nicker (*Caesalpinia bonduc*) green seed				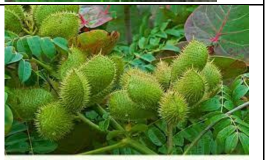
Oleander (*Nerium oleander*)				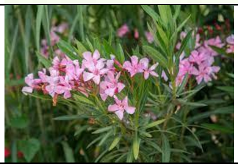

<u>Toxic Plant questions (cont.)</u>

Onion (*Alium cepa*) bulb			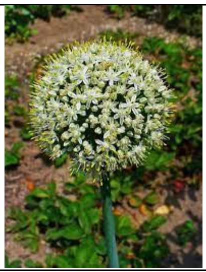
Painkiller bush (*Microtea debilis*)			
Periwinkle, ping wing white (*Catharantus roseus*)			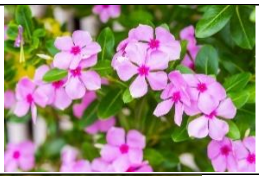
Pigeon pea (*Cajanus cajan*) root and leaves			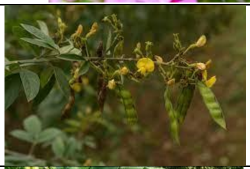
Plantain, plantin (*Musa paradisiaca*)			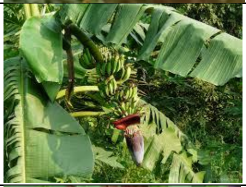
Pop bush (*Passiflora suberosa*)			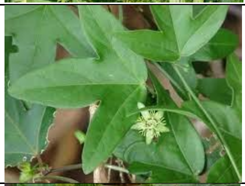
Prickly burr, fire blister bush (*Datura stramonium*) leaves and seeds			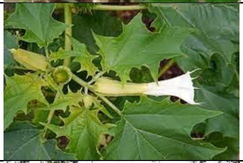
Rabbit food (bush), button weed, small leaf, mutton blam (*Leonotis nepetifolia*)			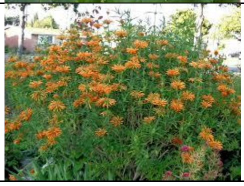

Ringworm bush, jumbee shuteye (*Cassia alata*)			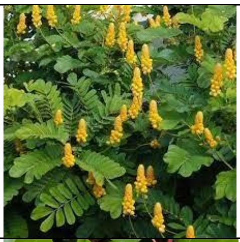
Roucou, knuckle seed (*Bixa Orellana*)			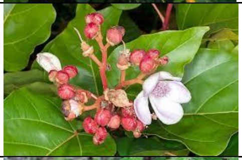
Sage (*Cordia curassavica*)			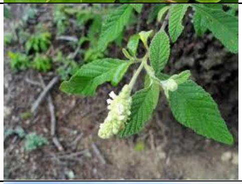
Sea side vine (*Ipomoea pes-caprae*)			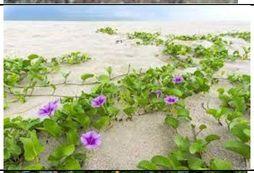
Shak shak (*Crotalaria retusa*)			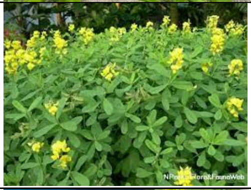
Shameful, Shamelady (*Mimosa pudica*)			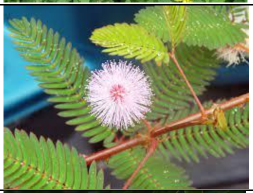
Sour orange (*Citrus aurantium*)			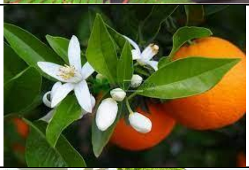
Stinging nettle (*Tragia volubilis*)			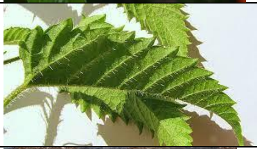
Thistle, sisle (*Argemone Mexicana*)			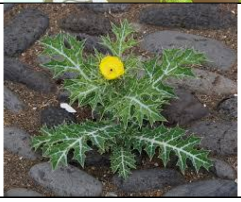

Tobacco (*Nicotiana tabacum*)			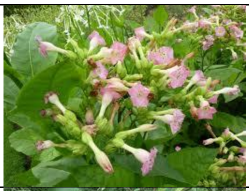
Wild eggplant, shushuba, pintop (*Solanum torvum*)			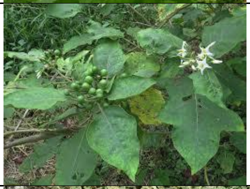
Wild pinda (*Tephrosis cinerea*)			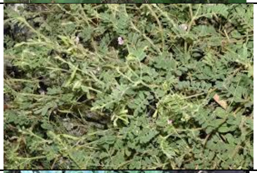
Wormwood (*Artemisia absinthium*)			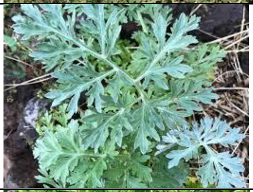
Worry wine, verry wine (*Stachytarpheta jamaicensis*)			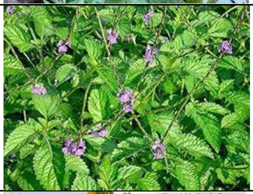
Yellow dad, dodder, love vine (*Cuscuta americana*)			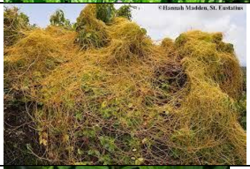
Yellow elder, yellow flower tree (*Tecoma stans*)			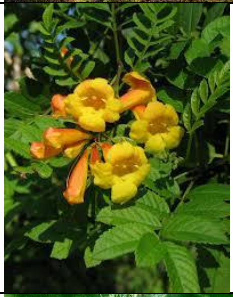
Castor Bean plant (*Ricinus communis*)			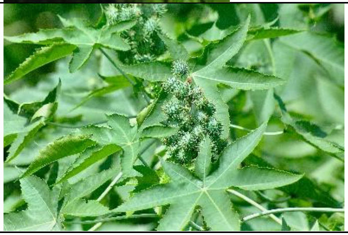
